# LRS1 phosphorylated in response to blue light regulates stomatal opening

**DOI:** 10.1007/s10265-026-01747-8

**Published:** 2026-08-10

**Authors:** Kohei Fukatsu, Yuki Hayashi, Keiko Kuwata, Toshinori Kinoshita

**Affiliations:** 1 Division of Biological Science, Graduate School of Science, Nagoya University, Nagoya, 464-8601 Japan; 2 Institute of Transformative Bio-Molecules (WPI-ITbM), Nagoya University, Nagoya, 464-8601 Japan

**Keywords:** *Arabidopsis thaliana*, Blue light, LRS1, Phosphorylation, Stomatal guard cells, *Vicia faba*

## Abstract

Blue light receptor phototropin is a protein kinase that initiates various blue light signaling pathways, including the phosphorylation and activation of plasma membrane (PM) H^+^-ATPase for stomatal opening. Activation of PM H^+^-ATPase drives K^+^ uptake via K^+^_in_ channels. This influx of K⁺ is electrically balanced by intracellular anions such as Cl⁻ and malate^2−^, increasing cellular osmotic potential and driving water influx and stomatal opening. Hypothesizing that unidentified phosphorylation events regulate this process, we performed phosphoproteomic analysis to elucidate the link between blue light signaling and stomatal opening. We found that blue light induces phosphorylation of Ser395 in *Vicia faba* LATERAL ROOT STIMULATOR 1 (VfLRS1) in guard cell protoplasts. VfLRS1 shows high similarity to *Arabidopsis thaliana* LATERAL ROOT STIMULATOR 1 (LRS1, AT3G05090), and the phosphorylation site is conserved as Ser393 in *A. thaliana* LRS1. Therefore, we investigated stomatal phenotype using a T-DNA-inserted knockout mutant of *A. thaliana* LRS1, *lrs1*. The *lrs1* mutant exhibited normal blue light-induced stomatal opening and normal blue light-induced phosphorylation of Thr948 of PM H^+^-ATPase (numbering according to AHA1) in guard cells. The *lrs1* mutant showed reduced starch accumulation during dark and red light illumination. Blue light-induced starch degradation was also severely suppressed in the *lrs1* mutant. The reduced starch accumulation in *lrs1* guard cells may have been associated with reduced levels of starch-derived metabolites, such as malate. We then examined blue light-induced stomatal opening in potassium gluconate (KGlu) buffer, in which most Cl^−^ was replaced by the poorly membrane-permeable gluconate anion. As expected, the *lrs1* mutant showed significantly reduced blue light-induced stomatal opening under this condition. Taken together, our results indicate that LRS1 plays important roles in starch dynamics and contributes to stomatal opening.

## Introduction

Stomata in the epidermis, each surrounded by a pair of guard cells, regulate gas exchange between leaves and the atmosphere through the stomatal pores. Light, CO_2_ and humidity are major factors affecting stomatal aperture (Inoue and Kinoshita 2017; Shimazaki et al. 2007). Stomatal opening enhances CO_2_ uptake for photosynthesis, transpiration and nutrient uptake in roots, resulting in increased plant growth and yields (Wang et al. 2014; Zeng et al. 2024). Light-induced stomatal opening in leaves is triggered by a combination of guard cell autonomous response and intercellular communication with mesophyll cells. Light that drives photosynthesis acts not only on chloroplasts in guard cells but also induces photosynthesis in mesophyll cells (Ando and Kinoshita 2018; Ando et al. 2022), thereby promoting stomatal opening via the mesophyll messengers, such as sucrose (Zait et al. 2025). In contrast, blue light triggers a guard cell autonomous response and directly induces stomatal opening.

Blue light is perceived by phototropins in guard cells; these phototropins phosphorylate the downstream kinase Blue Light Signaling 1 (BLUS1), resulting in activation (Kinoshita et al. 2001; Takemiya et al. 2013). This signaling pathway is modulated by other kinases such as Blue Light-dependent H^+^-ATPase Phosphorylation (BHP; Hayashi et al. 2017) and ultimately leads to the phosphorylation and activation of Thr948 in the plasma membrane (PM) H⁺-ATPase (numbering based on AHA1; Kinoshita and Shimazaki 1999; Palmgren 2001). Additionally, recent studies have shown that phosphorylation of Thr881 of PM H^+^-ATPase is also required for the full activation of H⁺-ATPase (Fuji et al. 2024; Hayashi et al. 2024). Of the protein kinases that regulate the phosphorylation status of PM H^+^-ATPase in guard cells, TOT3/MAP4K4 is thought to phosphorylate PM H^+^-ATPase Thr948 in guard cells in response to high-temperature stress (Xu et al. 2024). However, the protein kinase responsible for the phosphorylation of Thr881 remains unknown. As for protein phosphatases, Type 2C protein phosphatase clade D (PP2C.D) has been shown to mediate the dephosphorylation of both phosphorylated Thr948 and phosphorylated Thr881 in PM H^+^-ATPase in guard cells (Akiyama et al. 2022; Hayashi et al. 2024; Wong et al. 2021).

In parallel, phototropins may phosphorylate Convergence of Blue Light and CO_2_ (CBC) 1, suppressing the S-type anion channel, a key factor in stomatal closure, with the CBC1 homolog CBC2, and consequently promoting stomatal opening (Hiyama et al. 2017). However, the roles of CBC1 and CBC2 in guard cells remain controversial. CBC1 and CBC2 have also been reported to function as negative regulators of guard cell PM H^+^-ATPase phosphorylation in blue light-dependent stomatal opening (Hayashi et al. 2020).

Photosynthesis in guard cells contributes to the accumulation of starch granules in guard cell chloroplasts (Flütsch et al. 2022; Santelia and Lunn 2016). Accumulated starch has been shown to decrease with light exposure, accompanied by an increase in malate content (Schnable 1980). Malate functions as a counterion for K⁺ uptake during blue light-induced stomatal opening (Ogawa et al. 1978). Furthermore, blue light perceived by phototropins has been suggested to induce the degradation of starch granules in guard cell chloroplasts in association with PM H⁺-ATPase activation (Dang et al. 2024; Flütsch et al. 2020; Horrer et al. 2016). However, other studies have reported that blue light-induced stomatal opening can occur without substantial starch degradation in guard cells, suggesting species-dependent differences or the involvement of multiple metabolic pathways (Bahadar et al. 2025; Ogawa 1981; Tallman and Zeiger 1988). Thus, the relationship between blue light signaling and starch metabolism in guard cells remains incompletely understood.

In this study, to clarify the blue light signaling pathway in guard cells, we conducted a comprehensive phosphoproteomic analysis using guard cell protoplasts (GCPs) from *Vicia faba*. We found that the phosphorylation level of Ser395 in VfLRS1, an ortholog of LATERAL ROOT STIMULATOR 1 (LRS1, AT3G05090) in *Arabidopsis thaliana*, increased significantly in response to blue light. Genetic analysis using a knockout mutant of the ortholog in *A. thaliana* (*lrs1* mutant) revealed that the *lrs1* mutant showed normal blue light-induced stomatal opening and blue light-induced phosphorylation of PM H^+^-ATPase Thr948. On the other hand, a clear phenotype emerged under low-anion conditions. Based on these findings, we discuss the function of LRS1 in guard cells.

## Materials and methods

### Plant materials and growth conditions

*V. faba* (broad bean; Ryosai Issun) was cultivated hydroponically in a greenhouse at 20 °C under natural sunlight, as described previously (Hayashi et al. 2021; Kinoshita and Shimazaki 1999). *A. thaliana* plants were grown in soil under controlled growth-room conditions (16 h light/8 h dark, 50 µmol m^−2^ s^−1^, 24 °C, 55–70% humidity). The *lrs1* T-DNA insertion mutant (SALK_059570; AT3G05090) in the Col-0 background was obtained from the Arabidopsis Biological Resource Center (Ohio State University).

### Phosphoproteomics

GCPs from *V. faba* (500 µg protein) were isolated according to a previous method (Hayashi et al. 2021) and suspended in a suspension buffer (5 mM MES-NaOH [pH 6.0], 10 mM KCl, 0.4 M mannitol, and 1 mM CaCl_2_). After an initial 30 min dark adaptation, GCPs were subjected to one of three treatments: continued darkness for 30 min, exposure to strong red light (600 µmol m⁻^2^ s⁻^1^) for 30 min, or exposure to strong red light for 30 min followed by superimposed blue light (100 µmol m⁻^2^ s⁻^1^) for 1 min. Phosphoprotemic analysis was performed as described previously (Hayashi et al. 2024).

### Stomatal aperture measurements

The stomatal aperture in epidermal tissues was measured as described previously (Hayashi et al. 2011), with minor modifications. Epidermal fragments isolated from dark-adapted 4–5-week-old *A. thaliana* leaves were incubated in stomatal measurement buffer (5 mM MES-BTP, 10 mM KCl, 0.1 mM CaCl_2_, pH 6.5) or in potassium gluconate (KGlu) buffer (5 mM MES-BTP, 50 mM potassium gluconate, 0.1 mM CaCl_2_, pH 6.5). Epidermis fragments were illuminated for 3 h with either red light alone (50 µmol m⁻^2^ s⁻^1^) or with superimposed blue light (10 µmol m⁻^2^ s⁻^1^). Stomatal apertures were measured microscopically, with over 30 stomata analyzed per replicate.

### Immunohistochemical staining

Immunohistochemical staining was performed as described previously with minor modifications (Hayashi et al. 2011). Epidermal fragments were isolated from 4 to 6-week-old plants and treated with KGlu buffer. The fragments were then illuminated with red light (50 µmol m⁻^2^ s⁻^1^) for 20 min, after which a blue light pulse (10 µmol m⁻^2^ s⁻^1^) was applied for 2.5 min.

### Starch visualization with mPS-PI staining

mPS-PI staining was performed as described in Horrer et al. (2016) with modifications. Epidermal fragments peeled from leaves of 4–5-week-old plants were suspended in KGlu buffer and incubated either in the dark or under red light (50 µmol m⁻^2^ s⁻^1^) for 2 h. After red light illumination, blue light (10 µmol m⁻^2^ s⁻^1^) was superimposed on the red light for 20 min. Following light illumination, epidermal fragments were fixed in fixation solution (10% acetic acid, 50% methanol) and then treated with 1% periodic acid. The samples were subsequently stained with 0.1 mg ml^−1^ propidium iodide in Schiff’s reagent (100 mM sodium metabisulphite, 0.15 N HCl). Stained samples were then cleared with chloral hydrate solution (4 g chloral hydrate, 1 mL glycerol, 2 mL Milli-Q water) on glass slides for 1 day. The following day, 2–3 drops of Hoyer’s solution (15 g gum arabic, 100 g chloral hydrate, 8 mL glycerol, 25 mL Milli-Q water) were added, and the samples were covered with a cover glass. Samples were observed 2–3 days later using a confocal laser microscope. Starch signals were detected with an excitation wavelength of 488 nm and emissions collected at around 620 nm. Starch granule area was quantified using ImageJ.

## Results

### Blue light induces phosphorylation of Ser395 of VfLRS1 in guard cells from *V. faba*

Blue light-induced stomatal opening involves multiple protein phosphorylation events, including activation of the plasma membrane (PM) H⁺-ATPase. To identify phosphorylation changes induced by blue light, we performed phosphoproteomic analysis using GCPs from *V. faba*. This species was chosen because GCPs can be obtained at higher purity and in larger amounts (> 1 mg protein) than from *A. thaliana.* The *V. faba* expression database used in this study was constructed via RNAseq analysis (Hayashi et al. 2021). However, the release of the *V. faba* genome in 2023 enabled a more comprehensive interpretation of the proteomics data (Jayakodi et al. 2023).

**Fig. 1:**
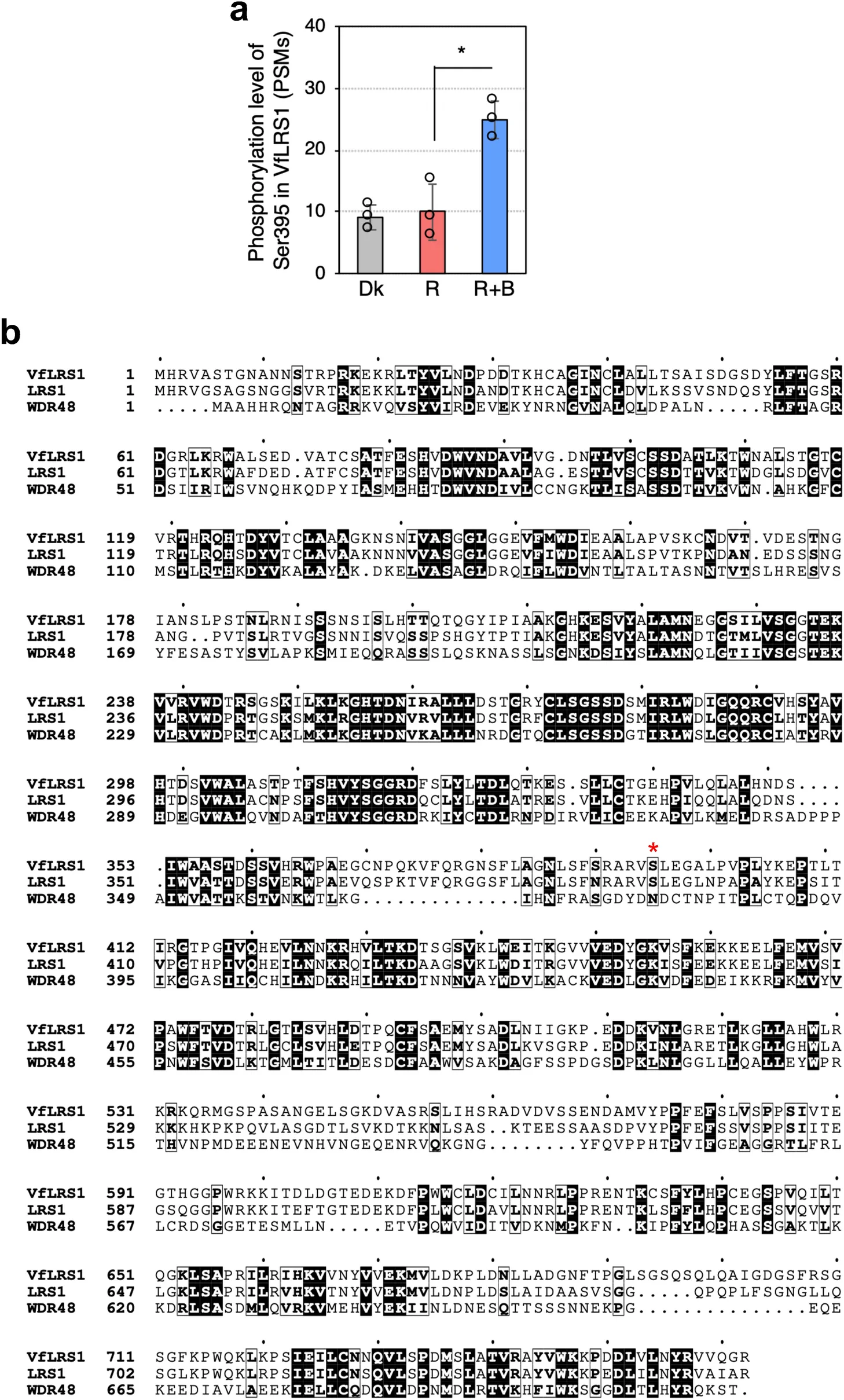
Blue light-induced phosphorylation of VfLRS1 in *Vicia faba* guard cell protoplasts (GCPs) and sequence alignment with LRS1 from *Arabidopsis thaliana* and Human WDR48. **a** Blue light-induced phosphorylation of Ser395 of VfLRS1 in *V. faba* GCPs. Peptide spectrum matches (PSMs) indicate the total number of identified peptide spectra matched for the VfLRS1 protein (*n* = 3). *V. faba* GCPs were illuminated with red light (600 µmol m^−2^ s^−1^; R) or incubated in the dark (Dk) for 30 min. After red light illumination, a weak pulse of blue light (100 µmol m^−2^ s.^−1^) was superimposed for 1 min (R+B). Means are presented ± SD, with asterisks (*) indicating significant differences (Student’s *t*-test, *p* < 0.05). **b** Alignment of deduced amino acid sequences of VfLRS1 (Vfaba.Hedin2.R1.5g183360.1) from *V. faba,* LRS1 from *A. thaliana* (AT3G05090), and human WDR48 (NP_001333154.1). Black boxes indicate strictly conserved amino acids and white boxes indicate similar amino acids among VfLRS1, AtLRS1, and Human WDR48. Periods (.) indicate gaps introduced to allow for optimal sequence alignment. The red asterisk indicates a conserved serine residue, which was phosphorylated by blue light in VfLRS1

We confirmed the blue light-induced phosphorylation events of phototropin (Vfphot1a and Vfphot1b), BLUS1, and Thr881 and Thr948 of PM H^+^-ATPase in *V. faba* GCPs (Table 1; Hayashi et al. 2024), suggesting that the Vicia GCPs used in this study can respond to blue light signals. Notably, we found that the phosphorylation level of Ser395 in Vfab028475.003 approximately doubled in response to a pulse of blue light superimposed on background red light in GCPs from *V. faba* (Table 1, Fig. 1a). Vfab028475.003, an ortholog of LATERAL ROOT STIMULATOR 1 (LRS1, AT3G05090) in *A. thaliana*, corresponds to Vfaba.Hedin2.R1.5g183360.1 (Faba Bean Genome Consortium; Jayakodi et al. 2023). We named Vfab028475.003 as VfLRS1; LRS1 encodes the DCAF (DDB1- and CUL4-associated factor) protein (Lee et al. 2008) and is involved in lateral root development (Lee et al. 2010).

**Table 1:** Phosphoproteomic analysis of Vicia GCPs illuminated with red and blue light

Contig	ID (Faba Bean Genome Consortium)	Phospho-site	PSMs								
			Rep.1			Rep.2			Rep.3		
			Dk	R	R + B	Dk	R	R + B	Dk	R	R + B
Vfphot1a	Vfaba.Hedin2.R1.6g032240.1	Ser350	0	0	9	0	0	5	5	5	13
Vfphot1b	Vfaba.Hedin2.R1.1g037520.1	Ser350	0	0	15	0	0	10	0	0	11
BLUS1	Vfaba.Hedin2.R1.5g011880.1	Ser348	8	19	65	11	13	47	13	13	67
VHA1	Vfaba.Hedin2.R1.4g215240.1	Thr881	10	98	136	11	26	65	11	22	70
Unnamed isoform	Vfaba.Hedin2.R1.1g418440.1		0	24	35	0	1	10	1	0	16
VHA1/Unnamed isoform	Vfaba.Hedin2.R1.1g418440.1 Vfaba.Hedin2.R1.4g215240.1	Thr948	31	99	136	50	49	89	44	51	113
VfLRS1	Vfaba.Hedin2.R1.5g183360.1	Ser393	11	6	26	7	15	22	9	9	25

Peptide spectrum matches (PSMs) of each phospho-peptide from Vfphot1a, Vfphot1b, BLUS1, VHA1, unnamed isoform, and VfLRS1 are indicated. ID indicates the most similar protein in the Faba Bean Genome Consortium. The phospho-site numbers correspond to the amino acid number of the Arabidopsis homolog protein. Results are presented for each of three experimental replicates

Figure 1b shows the alignment of deduced amino acid sequences of VfLRS1 and *A*. *thaliana* LRS1. The deduced proteins consist of 762 and 753 amino acids, with predicted molecular masses of 83.5 and 82.2 kD, respectively. VfLRS1 and *A. thaliana* LRS1 contain conserved domains typical of the WD repeat protein (amino acids 34–365 for VfLRS1 and 34–323 for *A. thaliana* LRS1; Lee et al. 2008). The full-length VfLRS1 protein shares 70.8% of its amino acids with *A*. *thaliana* LRS1 protein, including conserving the serine residue (Ser393, corresponding to Ser395 in VfLRS1) that is phosphorylated in response to blue light. These results suggest that VfLRS1 and *A. thaliana* LRS1 are orthologs. VfLRS1 also showed sequence similarity to human WD repeat (WDR) 48, but the blue light-induced phosphorylation site identified in VfLRS1 was not conserved in WDR48 (Fig. 1b).

### The *lrs1* mutant shows normal blue light-induced stomatal opening

**Fig. 2:**
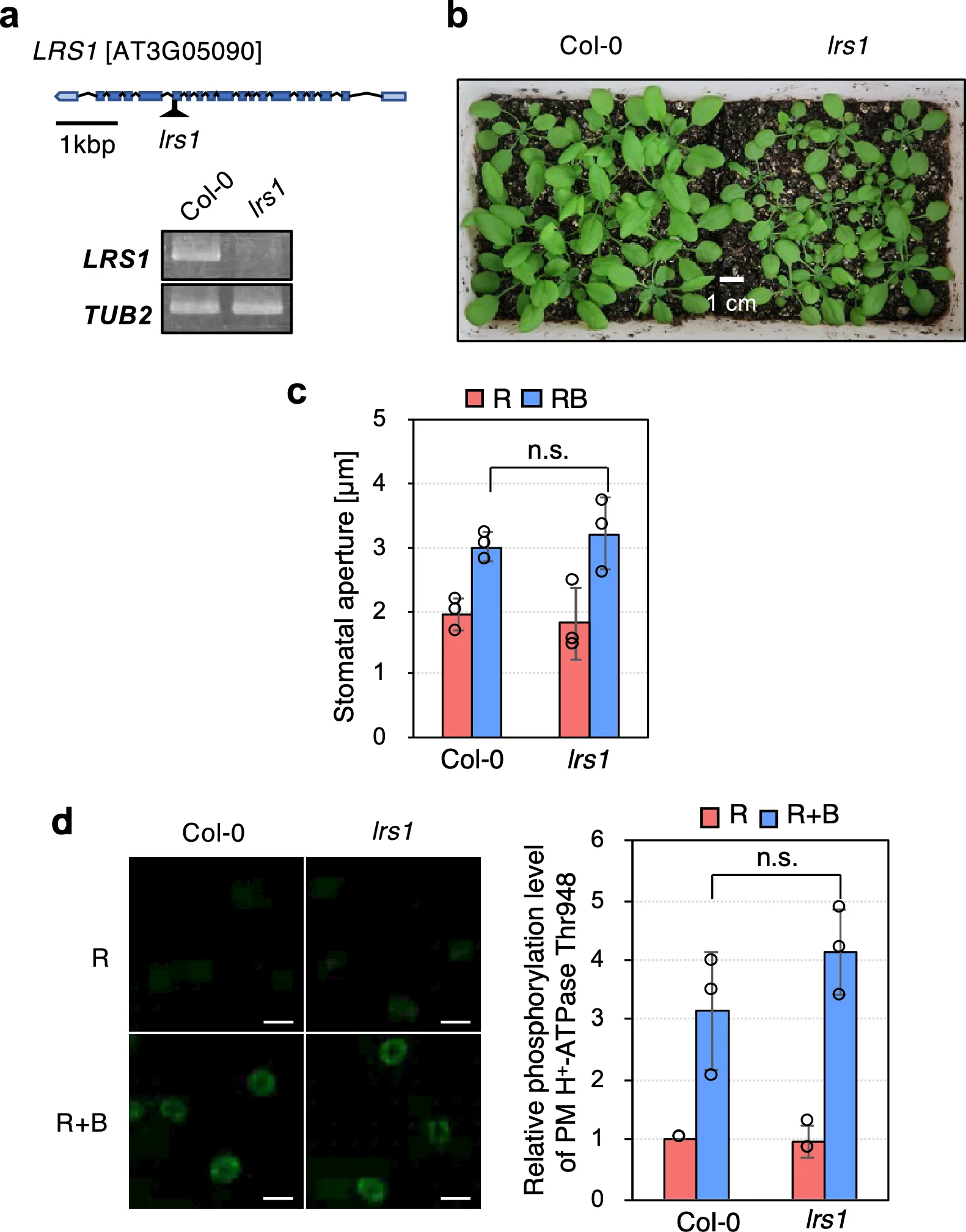
Stomatal phenotypes of *lrs1* in response to blue light. **a** The position of T-DNA in the *LRS1* gene and *LRS1* gene expression detected by RT-PCR in plant leaves. Blue boxes and interconnecting black lines indicate exons and introns, respectively. The light blue box indicates UTR. **b** Plant growth phenotypes of four-week-old *lrs1* mutants and Col-0. **c** Blue light-induced stomatal opening in *lrs1* mutants (*n* = 3). Isolated epidermal peels were illuminated in stomatal measurement buffer for 3 h. Light conditions were red light only (50 µmol m^−2^ s^−1^; R) or both red and blue light (red light: 50 µmol m^−2^ s^−1^, blue light: 50 µmol m^−2^ s^−1^; RB). Means are presented ± SD, with statistical significance determined via Student’s *t*-test (n.s.: not significant). **d** Immunohistochemical detection of blue light-induced phosphorylation of Thr948 of PM H^+^-ATPase in guard cells in response to blue light (*n* = 3). Isolated epidermal peels were illuminated with red light (50 µmol m^−2^ s^−1^) in KGlu buffer for 30 min. After red light illumination for 30 min (R), blue light (10 µmol m^−2^ s.^−1^) was superimposed for 2.5 min (R + B). The photograph represents a typical signaling image; scale bars indicate 20 µm. Means are presented ± SD, with statistical significance determined via Student’s *t*-test (n.s.: not significant)

Given the difficulty of performing genetic analysis in *V. faba*, we analyzed stomatal phenotypes in the *A*. *thaliana lrs1* knockout mutant to investigate the role of LRS1 in guard cells (Fig. 2a). Figure 2b shows photographs of 4-week-old plants. Compared with Col-0, *lrs1* plants exhibited slightly reduced growth. The *lrs1* mutant exhibited normal blue light-induced stomatal opening in stomatal measurement buffer (Fig. 2c) and normal blue light-induced phosphorylation of PM H^+^-ATPase Thr948 similar to that of Col-0 (Fig. 2d). These results suggest that *lrs1* retains a normal blue light response.

### The *lrs1* mutant shows reduced starch accumulation in guard cells

**Fig. 3:**
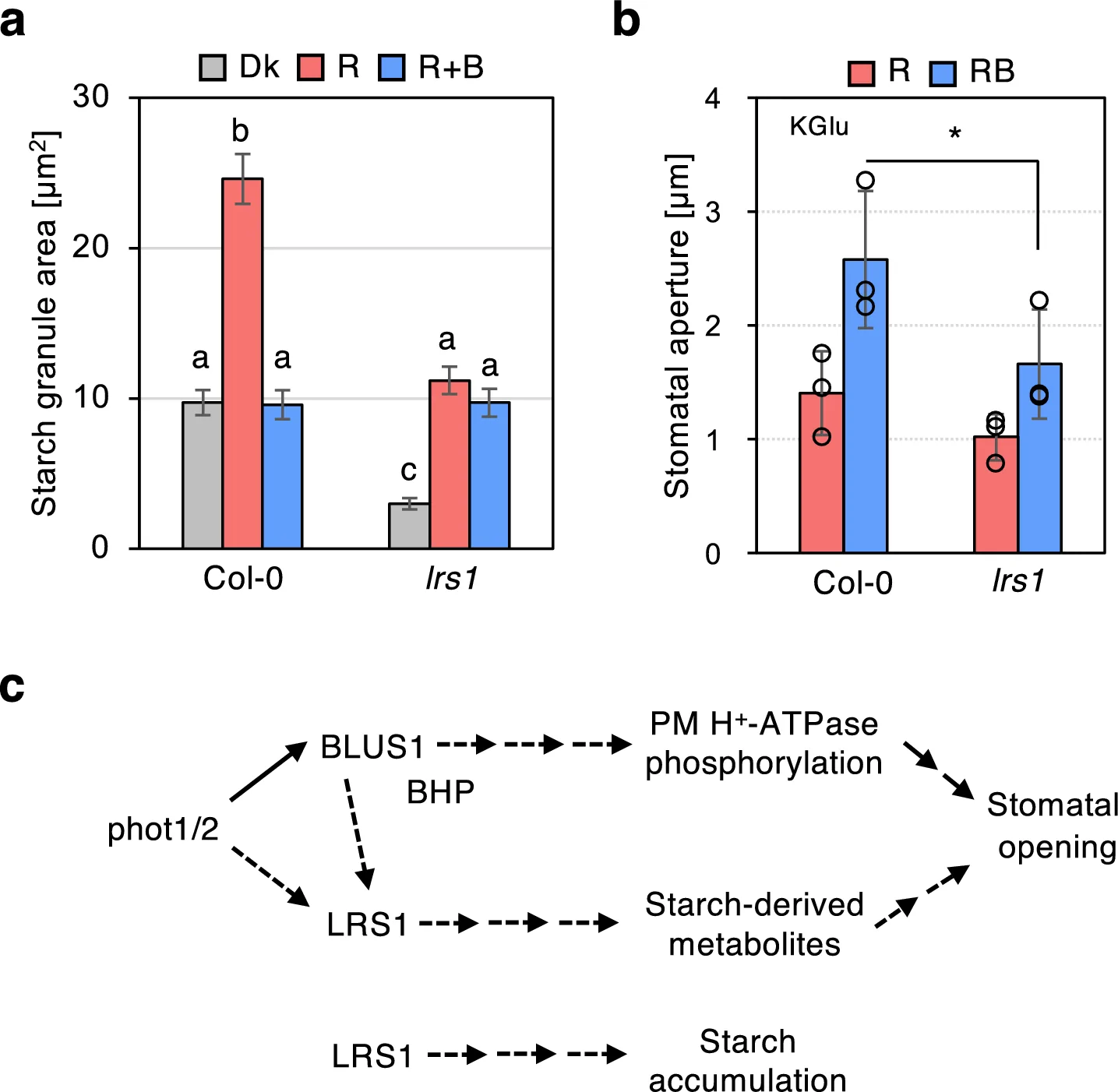
Phenotypic properties of the *lrs1* mutant. **a** Starch dynamics in guard cells in response to light. Isolated epidermal peels were illuminated with red light (50 µmol m^−2^ s^−1^; R) or incubated in the dark (Dk) for 2 h. After red light illumination, blue light (10 µmol m^−2^ s^−1^) was superimposed for 15 min (R + B). Graphs present the means ± SE of three biological replicates of > 99 individual guard cells obtained from three independent experiments. Different letters indicate significant differences (ANOVA with Tukey’s test, *p* < 0.05). **b** Blue light-induced stomatal opening in *lrs1* mutants (*n* = 3). Isolated epidermal peels were illuminated in KGlu buffer for 3 h. Light conditions were red light only (50 µmol m^−2^ s^−1^; R) or both red and blue light (red light: 50 µmol m^−2^ s^−1^, blue light: 10 µmol m^−2^ s^−1^; RB). Means are presented ± SD, with asterisks (*) indicating significant differences (Student’s* t*-test, *p* < 0.05). **c** Schematic model of LRS1 in blue light-induced stomatal opening. Dotted arrows indicate unidentified steps

We next checked the dynamics of starch accumulation and degradation in guard cells via mPS-PI staining (Fig. 3a). In Col-0 guard cells, starch accumulated during 2 h of red light illumination and was subsequently degraded after 15 min of blue light illumination superimposed on red light. In contrast, the starch content in *lrs1* guard cells was significantly lower than that in Col-0 in the dark. Although red light induced a slight increase in starch content after 2 h of illumination, starch accumulation in *lrs1* remained less than half that observed in Col-0. Moreover, we observed no starch degradation by blue light illumination. These results suggest that *lrs1* mutation influences starch dynamics, such as deficiency of starch accumulation or constant activation of starch degradation, in guard cells. Given these results, we used potassium gluconate (KGlu) buffer, in which most Cl^−^ was replaced by the poorly membrane-permeable gluconate anion, to investigate stomatal aperture in *lrs1* and found that blue light-induced stomatal opening was significantly suppressed in the *lrs1* mutant (Fig. 3b). These results suggest that the reduced starch content in *lrs1* guard cells may limit the availability of endogenous anions required for blue light-induced stomatal opening under Cl⁻-deficient conditions.

## Discussion

In this study, phosphoproteomic analysis of GCPs from *V. faba* identified LRS1 as a novel factor that is phosphorylated in guard cells in response to blue light (Fig. 1a). Analysis using the *lrs1* mutant of *A. thaliana* revealed that, although blue light-induced stomatal opening and phosphorylation of Thr948 of the plasma membrane H⁺-ATPase—events crucial for blue light activation—were normal in the mutant, light-induced starch accumulation was reduced (Figs. 2, 3a). Notably, the *lrs1* mutant showed reduced light-induced stomatal opening under reduced anion conditions (Fig. 3b). Therefore, it is possible that the levels of starch-derived metabolites, such as malate, produced during subsequent starch degradation were also decreased, as malate plays a crucial role in light-induced stomatal opening as a counterion for K⁺ (Ogawa et al. 1978).

LRS1 is a WD repeat (WDR) protein known as a DDB1- and CUL4-associated factor. The DDB1–CUL4 complex functions as a ubiquitin ligase and has been shown to regulate diverse cellular processes, including protein degradation, activity regulation, chromatin control, and DNA repair signaling (Olazabal-Herrero et al. 2018). Within this complex, LRS1 is thought to act as a substrate-recognition protein (Lee et al. 2008). A previous study suggested that LRS1 is involved in lateral root development induced by auxin accumulation through CUL4-mediated protein degradation by the 26S proteasome (Lee et al. 2010). Based on this, it is possible that LRS1 influences starch accumulation and starch-derived metabolism through 26S proteasome-mediated protein degradation, ultimately contributing to stomatal opening (Fig. 3c).

WDR proteins possess multiple blades forming a ß-propeller structure, and proteins containing seven blades are generally thought to exhibit higher structural stability (Mishra et al. 2012; Schapira et al. 2017). VfLRS1 and LRS1 show high sequence similarity to the human homolog WDR48 (Yin et al. 2015) (Fig. 1b). WDR48 (also known as USP1-associated factor 1, UAF1) is a WD-repeat protein that activates several deubiquitinating enzymes, including USP1, USP12, and USP46. However, the phosphorylated residue identified in this study is not conserved in human WDR48 (Fig. 1b), suggesting that plant LRS1 may be subject to plant-specific regulatory mechanisms. Future studies are needed to determine how this phosphorylation affects the function of LRS1. Although LRS1 is phosphorylated in response to blue light, the upstream kinase responsible for this phosphorylation remains unidentified (Fig. 3c). It is currently unclear whether the kinase is phototropin (phot) itself or downstream kinases such as BLUS1 or BHP, and identifying the upstream kinase will be an important subject for future investigation.

In summary, our study suggests that blue light-induced phosphorylation of the newly identified protein LRS1 confers at least two functions in guard cells. One is the regulation of starch accumulation in guard cell chloroplasts, although the exact mechanism by which LRS1 is involved in starch accumulation remains unclear. The other is the production of starch-derived metabolites. In terms of timing, blue light-induced phosphorylation of Thr948 of the plasma membrane H⁺-ATPase, which corresponds to its activation, occurs within 30 s of the onset of blue light (Kinoshita and Shimazaki 1999), and blue light-induced phosphorylation of VfLRS1 is also triggered within 1 min (Fig. 1a). LRS1 may therefore contribute to efficient stomatal opening by rapidly initiating the production of starch-derived metabolites in response to blue light (Fig. 3d). Future studies examining the levels of starch-derived metabolites, including malate, in guard cells of the *lrs1* mutant are needed to further clarify this mechanism.
